# Correlation and Interchangeability of Amyloid, Tau, and Glucose Metabolism PET in Mild Cognitive Impairment and Alzheimer: A Review

**DOI:** 10.3390/brainsci15121271

**Published:** 2025-11-26

**Authors:** Emile Balot, Stefaan Vandenberghe, Tim Van Langenhove, Valerie De Meulenaere, Yves D’Asseler, Donatienne Van Weehaeghe

**Affiliations:** 1 Department of Radiology and Nuclear Medicine, Ghent University Hospital, 9000 Ghent, Belgium; emile.balot@uzgent.be (E.B.);; 2 Medical Image and Signal Processing, Faculty of Engineering, Ghent University, 9000 Ghent, Belgium; 3 Cognitive Center, Department of Neurology, Ghent University Hospital, 9000 Ghent, Belgium

**Keywords:** Alzheimer’s disease, amyloid PET, tau PET, FDG PET, A/T/N, multi biomarker, early phase PET, deep learning

## Abstract

Positron emission tomography (PET) allows for minimally invasive in vivo localization of amyloid and tau deposition, and visualization of glucose metabolism using amyloid PET, tau PET, and FDG PET. Clinically, these scans are used to determine A, T, and N (amyloid-β plaques, tau tangles, and neurodegeneration) status in Alzheimer’s disease. In light of the recent anti-amyloid therapies, determination of the A, and the associated T and N status is increasingly important. This review explores the potential of a single PET scan to define multiple biomarkers. A literature search using the PubMed database and an additional citation search using Google Scholar identified 76 relevant publications up to 30 July 2025. Original work reporting amyloid, tau or FDG PET to determine two or more ATN-related biomarkers were included. Non-English, animal, and non-dementia related studies were excluded. For each study, quantitative outcomes such as correlations and ROC AUC scores were extracted and compared. Early phase amyloid and tau PET (*n* = 58) were consistently found to be good indicators of N status with a median (IQR) correlation of 0.82 (0.76–0.86). Limited research (*n* = 7) was performed for amyloid or tau PET to infer both A and T status, with tau-based studies having slightly higher ROC AUC scores (0.88–0.99) than amyloid-based studies (0.84–0.9). Initial results are promising (median ROC AUC scores of 0.88 (0.81–0.96)) but need to be validated. FDG PET was found to be less accurate for A or T status (*n* = 12) prediction (median ROC AUC scores of 0.83 (0.80–0.87)). Among the modalities, tau PET seems to be the most promising candidate for a single tracer approach to predict all three biomarkers.

## 1. Introduction

Alzheimer’s disease (AD) is a neurodegenerative disease characterized by the accumulation of amyloid-β plaques and tau neurofibrillary tangles and is the most common cause of dementia. AD is quickly becoming one of the largest challenges in healthcare [1], with the prevalence of dementia projected to nearly double in Europe [2] and almost triple worldwide by 2050 [3].

Accurate diagnosis of the underlying cause of dementia is essential for determining the correct treatment options and is in line with the new biological concept of the NIA-AA [4]. The two core biomarkers of the AD neuropathologic change for diagnosis and staging are amyloid-β (A) and tau (T). Different in the newest criteria is that T status is has been divided into 2 subcategories: T1 which becomes abnormal at a similar time as A status and in which phosphorylated/secreted AD tau is measured, and T2 which becomes abnormal later in the disease, is more closely linked to the onset of symptoms, and in which AD tau proteinopathy is measured. So, A status is determined using fluid assays (cerebrospinal fluid (CSF) or plasma), in which Aβ proteinopathy or phosphorylated/secreted AD tau (T1) is measured, or amyloid PET (positron emission tomography). T status is determined using fluid assays in which AD tau proteinopathy is measured or tau PET scans. Previously, neurodegeneration (N) was part of the main biomarkers in the widely used ATN framework [5,6]. N status is, however, non-specific for AD and therefore no longer considered part of the core biomarkers in the new framework [4]. Still, assessing the N status is invaluable in clinical practice for staging, prognosis, identifying copathologies, and as an indicator of treatment effect. N status can be evaluated by hypometabolism patterns of [18F]-fluorodeoxyglucose (FDG) PET, atrophy on structural MRI (magnetic resonance imaging), and neurofilament light chain (Nfl) in fluid assays (CSF or plasma).

With anti-amyloid therapies for early AD becoming available, correct diagnosis using the biological concept is indispensable. In Europe, there are currently two approved anti-amyloid antibodies, Lecanemab [7,8] and Donanemab [9]. The latter used both amyloid and tau PET as secondary endpoints in its phase 3 clinical trial [10]. However, performing multiple PET scans to determine the A, T, and N status, respectively, results in an elevated radiation dose, time investment, and economic burden for the patient and the healthcare system.

A, T, and N status are inherently correlated due to the biological interactions of the pathological AD pathway [11,12]. Previous studies demonstrated hypometabolism patterns visualized on FDG PET are closely linked to perfusion measures as metabolism reflects a combination of neuronal activity and perfusion [13,14]. Additionally, amyloid and tau PET are assumed to be a downstream effect of one another and therefore it is hypothesized that by using deep learning (DL) or other tools, amyloid or tau PET could be used to predict both A and T status. It is thus proposed that by dynamic or dual-phase amyloid or tau PET scanning, A, T, and N could all be obtained in a single PET exam.

This review will summarize the current literature on how single PET imaging can determine multiple biomarkers by using different time windows, region-specific analyses, kinetic modelling strategies, or artificial intelligence (AI).

Similarly to PET, MRI has also been explored to predict multiple biomarkers. Some recent studies have investigated DL methods to predict amyloid positivity using MRI [15,16] or for the synthesis of amyloid or tau PET images from MRI [17,18]. However, given their limited clinical applicability, these methods are outside of the scope of this review. Instead, this review article will focus on how single PET imaging can be leveraged to extract multiple AD biomarkers, thereby reducing the number of PET modalities necessary. In particular, the review will explore how FDG, amyloid, and tau PET are correlated and whether they can be used interchangeably in MCI and AD.

## 2. Materials and Methods

**Figure 1:**
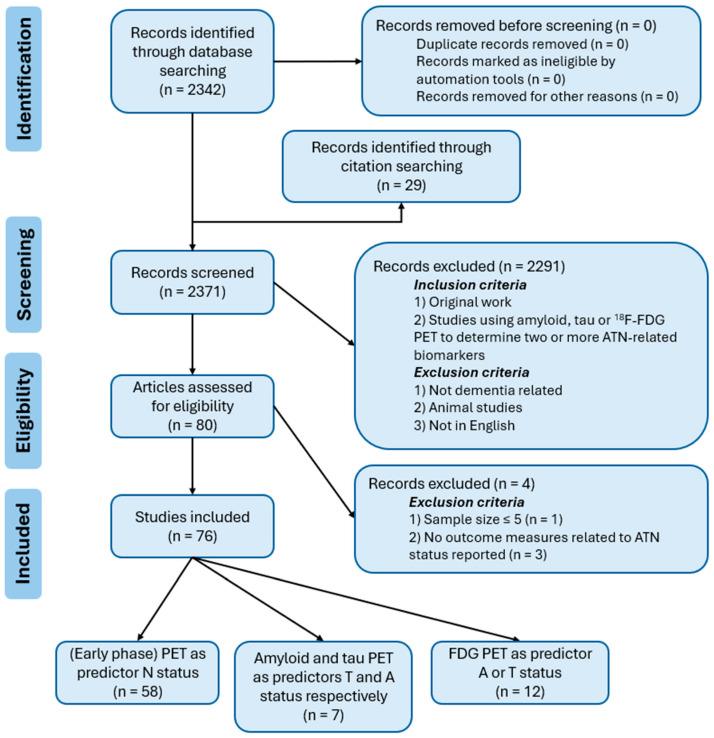
PRISMA flow diagram. PubMed database search on the 30 July 2025, citation searching using Google Scholar.

The focus of this review is in highlighting the variety of novel methods emerging in this field. Given the limited and heterogenous nature of the existing literature, a strict systematic review structure was not feasible. Nonetheless, the systematic reviews and meta-analyses (PRISMA) guidelines [19] were still largely followed to strengthen the transparency and reproducibility of the review (Figure 1).

To gather the relevant literature, a search was performed on the 30 July 2025 using the PubMed database. The following query was used: (surrogate OR predict OR correlate) AND (tau PET OR amyloid PET) AND (Alzheimer* OR dementia) NOT (plasma) NOT (mouse). The query included MeSH terms that were automatically added by PubMed. The detailed search query can be found in the Supplementary Material. No date restriction was set, all available studies up to 30 July 2025 were included in the search. No automation tools were used or duplicates removed before screening. To identify the additional relevant literature, we manually explored both backward citations (reference lists) and forward citations (subsequent papers citing the studies) using Google Scholar. One reviewer conducted the search and selection process, with any uncertainties resolved through discussion with a second reviewer.

The database search yielded 2342 articles and 29 more were identified through citation searching. Studies were screened for eligibility based on the following criteria: (1) original work, (2) studies using amyloid, tau or FDG PET to determine two or more ATN-related biomarkers. Exclusion criteria were as follows: (1) not dementia related, (2) animal studies, and (3) not written in English. Screening was performed using the articles’ abstract. To identify emerging trends, a small number of non-peer-reviewed preprints (*n* = 1) or abstracts (*n* = 1) were included because they represent very recent studies that are the first and only to introduce certain novel approaches and these will be clearly identified when discussed.

The first screening yielded 80 eligible articles. After reviewing the methods and results, 4 studies were excluded based on the following criteria: (1) insufficient sample size (*n* ≤ 5), and (2) no outcome measures related to ATN status reported. Study quality was assessed using the Quality Assessment of Diagnostic Accuracy Studies-2 (QUADAS-2) tool [20]. The tool assesses risk of bias and applicability concerns in four domains: patient selection, index test, reference standard, and flow and timing. Quality assessment results are summarized in Appendix A.

The final selection comprised a total of 76 articles, divided into three groups based on their primary focus: (1) (early phase) amyloid or tau PET to determine N status, (2) amyloid and tau PET to predict T and A status, respectively, and (3) FDG PET to predict A or T status. This resulted in 58, 7, and 12 studies for each group, respectively (one study is included in both group 2 and 3). For each group, a table listing the included publications and relevant features is provided (the tables in Section 3).

## 3. Results

### 3.1. Quality Assessment

**Figure A1:**
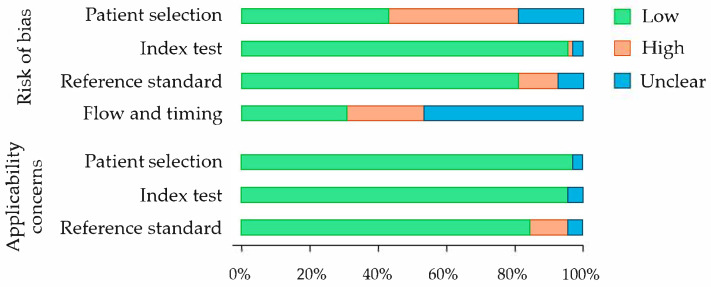
Graphical representation of QUADAS-2 results of the 76 included studies.

Quality assessment was performed for all included studies using the Quality Assessment of Diagnostic Accuracy Studies-2 (QUADAS-2) tool [20]. Summarized results are represented in tabular (Table A1) and graphical (Figure A1) form in Appendix A.

Patient selection was rated ‘high’ for a large number of studies, mostly because of retrospective studies or when using research databases such as ADNI. Similarly, a large number of studies were rated ‘unclear’ when it was not adequately described how enrolment was performed. The overall bias for the index test and reference standard categories was low. Flow and timing were rated ‘high’ for a large number of studies when the time between the index test (e.g., early phase PET) and reference standard (e.g., FDG PET) were 6 months or higher and ‘unclear’ if not mentioned. Applicability concerns were rather low overall, because the included studies already passed eligibility criteria during the search.

### 3.2. Amyloid and Tau PET as Predictors of N Status

#### 3.2.1. General Characteristics

**Figure 2:**
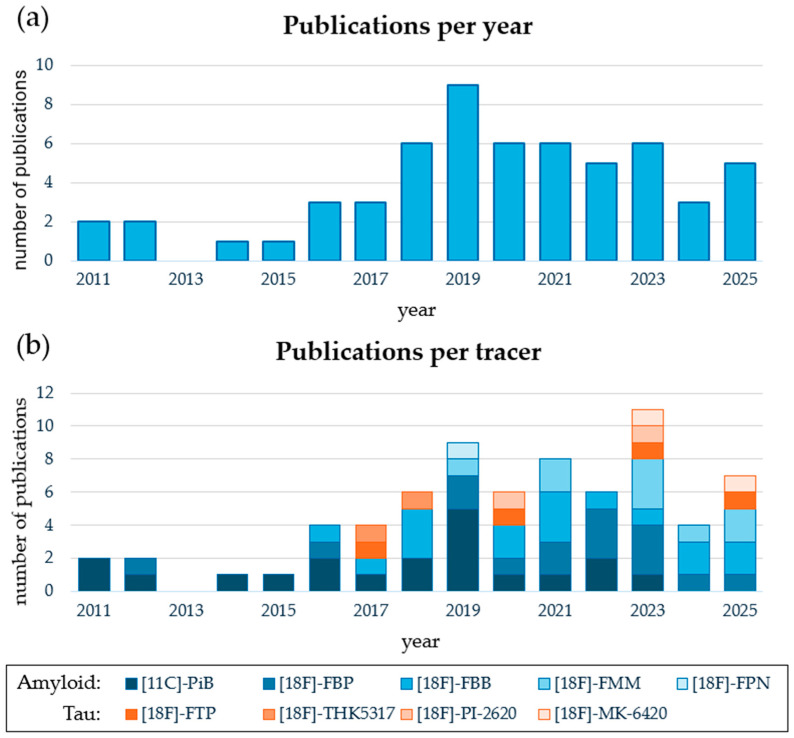
General characteristics of studies that use amyloid or tau PET as predictors of N status: (**a**) number of publications per year; (**b**) number publications per radiotracer per year, amyloid PET tracers are represented by shades of blue and tau PET tracers by shades of orange. Because some publications use multiple tracers, the counts can be different between both graphs.

A total of 58 studies investigated the use of amyloid or tau PET as predictors of neurodegeneration (N) status. Characteristic features are summarized in Table 1. Figure 2a shows the distribution of studies per year. Interestingly, the number of publications stayed stagnant after 2019. However, there has been a clear increase in the variety of radiotracers and methods explored (Figure 2b). More than 80% of included studies used amyloid PET. The most frequently reported amyloid radiotracer was [11C]-Pittsburgh compound B ([11C]-PiB), followed by [18F]-florbetaben ([18F]-FBB), [18F]-florbetapir ([18F]-FBP), [18F]-flutemetamol ([18F]-FMM), and a single study used [18F]-florapronol ([18F]-FPN). Comparatively, the number of tau PET studies was rather limited with most papers reporting [18F]-flortaucipir ([18F]-FTP), and then in equal, smaller numbers [18F]-PI-2620, [18F]-THK5317, and [18F]-MK-6420. Sample sizes ranged from around 10 to 230 participants consisting of cognitively unimpaired (CU), mild cognitive impaired (MCI), and dementia patients. Most studies focused on patients along the AD spectrum, but also patients suffering from frontotemporal dementia (FTD), Parkinson’s disease dementia, primary progressive aphasia, Lewy body dementia, and others were included.

#### 3.2.2. Analytical Approaches and Findings

Most studies compared their surrogate N measures against FDG PET, which is considered the gold standard for assessing cerebral metabolism. A smaller number of studies compared to perfusion images obtained by [15O]-H_2_O PET (gold standard for perfusion), ASL MRI or [Tc-99m]-ECD SPECT. Studies that did not have access to FDG or perfusion scans compared their results to early phase amyloid or tau PET scans that were previously shown to correlate strongly with FDG PET.

Practically all studies reported high visual similarities between amyloid or tau PET-based N-surrogates and FDG PET. Z-score maps demonstrated disease-specific patterns that closely resembled those seen on FDG PET. Quantitatively, both volume-of-interest (VOI) based analyses and voxel-wise analyses showed consistently high correlations across radiotracers, early phase time windows, and kinetic modelling approaches. Cortical regions were highly correlated, with occipital regions generally the weakest and parietal regions the strongest. Subcortical regions, such as the hippocampus and thalamus, were also correlated with FDG PET, although to a lesser degree than cortical areas.

Correlation coefficients can be biassed in case-controls designs, where the inherent difference between healthy control and advanced dementia patient drives the correlation. The risk of bias due to patient selection was assessed and is reported in Table A1. Several studies compared performance across disease stages (CU, MCI, or dementia) [26,28,29,48,51] and overall reported no significant differences in correlations between each group. When stratified by amyloid status, most studies similarly found no significant differences between amyloid-positive and amyloid-negative groups. Most reported slightly higher (but non-significant) correlations for amyloid-positive groups, although this could be attributed to more severe hypometabolism variations in amyloid-positive cases resulting in higher correlations [26,48,54,69]. One study compared tau-positive and tau-negative groups and found similar results [27]. Importantly, strong correlations between early phase amyloid or tau PET and neurodegeneration measures were not restricted to Alzheimer’s disease: patients with frontotemporal dementia (FTD) [22,62], Parkinson’s disease dementia (PDD) [23], Lewy body dementia (LBD) [77], and other non-AD pathologies also showed comparable results. Previous studies demonstrated hypometabolism patterns visualized on FDG PET are closely linked to perfusion measures as metabolism reflects a combination of neuronal activity and perfusion [13,14]. As early frames of highly lipophilic tracers are driven by the delivery rate, they behave as perfusion tracers in this time window and are minimally influenced by their late-phase binding characteristics. Thus, the amyloid or tau status were not expected to impact the correlation. Furthermore, even for disparate diseases the early perfusion-like images from amyloid or tau PET seemed to be a valid neurodegeneration surrogate.

Diagnostic performance was likewise similar to FDG PET. High AUC values were reported for differentiating AD patients from healthy controls and showed comparable discriminative power to FDG PET. Moreover, correlations with cognitive measures such as MMSE scores were robust and in the same range as those for FDG PET.

Three main approaches have been explored for representing neuronal injury using amyloid or tau PET scans. The most widely used and simplest surrogate is the early phase image, obtained by averaging a selection of early frames from a dynamic scan. A second method was using relative perfusion parametric maps (R1), which are obtained by kinetic modelling. More recently, artificial intelligence (AI) techniques have been applied to predict N status or even generate synthetic FDG PET images.

#### 3.2.3. Early Phase PET

Obtaining an early phase PET image that reliably reflects cerebral perfusion requires careful selection of the time window. In the first minutes after tracer injection, the signal primarily represents tracer delivery and transfer across the blood–brain barrier, whereas later frames increasingly reflect tracer uptake and binding equilibrium. As a result, shorter time windows provide images closer to true perfusion; however, the image quality is also significantly reduced when taking shorter scans.

**Figure 3:**
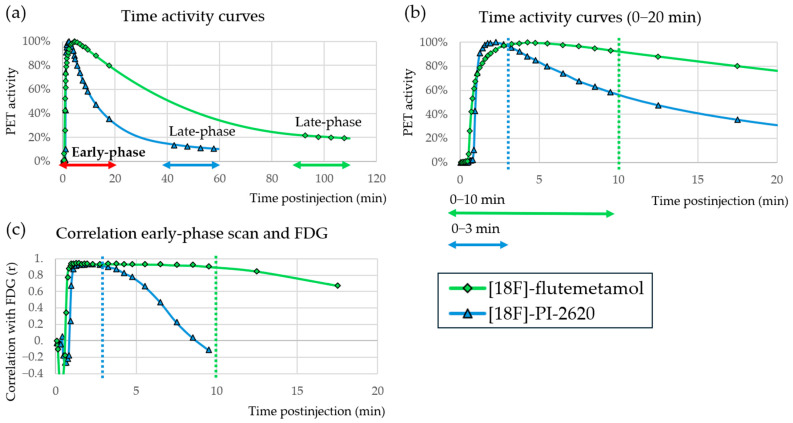
Demonstrative time activity curves (mean of cortical regions) and correlation plots of [18F]-flutemetamol and [18F]-PI-2620 obtained from in-house data. The dotted lines indicate the correlation drop-off point. (**a**) Time activity curve of the full scan duration. Early phase and late-phases are indicated. (**b**) Time activity curve of the early phase, with an indication of the most frequently used cut-off points. (**c**) Correlation graph between early phase frames and FDG.

Per radiotracer the most commonly used time window is: 1–8 min for [11C]-PiB, 0–10 min for [18F]-FBB, 0–5 min/1–6 min for [18F]-FBP, 0–10 min for [18F]-FMM, 0–10 min for [18F]-FPN, 0–10 min/1–6 min for [18F]-FTP, 0.5–2.5 min for [18F]-PI-2620, 0–3 min for [18F]-THK5317, and 0–3 min for [18F]-MK-6420. Notably, tau tracers generally have shorter time windows than amyloid tracers. This can be explained by the differing kinetic properties as represented by the time activity curves in Figure 3. For amyloid PET, the activity reaches its peak at around 4 min, while for tau PET the activity peak is much sharper and reaches its peak closer to 2 min. Both tracers reach the highest correlation (>0.9) with FDG PET around 1 min. The amyloid tracer then keeps a relatively high correlation for the first 10 min, while the tau tracer shows a rapidly declining correlation after about 3 min. For the time frames mentioned, all radiotracers achieved similar VOI-based correlation scores against FDG PET (averaged across studies): 0.76 for [11C]-PiB, 0.82 for [18F]-FBB, 0.80 for [18F]-FBP, 0.83 for [18F]-FMM, 0.83 for [18F]-FPN, 0.85 for [18F]-FTP, 0.76 for [18F]-PI-2620, 0.84 for [18F]-THK5317, and 0.84 for [18F]-MK-6420 (against [15O]-H_2_O instead). Interestingly, studies that explored ultrashort time frames (e.g., 0–1 min) [39,69] reported higher correlations (up to 0.92) than their longer counterparts. It should be kept in mind that differences can be partly attributed to factors such as patient selection, sample size, PET scanner type or timing of dynamic scan start. The quality of the early phase PET scan largely depends on the sensitivity of the PET scanner, availability of time-of-flight and reconstruction parameters used. Smoothing, reconstruction algorithm and number of iterations and subsets (causes sharpness of the images with increasing numbers, which can alter the signal to noise ratio) affect image quality, especially as longer acquisition times are not possible due to the limited time frame with perfusion-like characteristics (Figure 3). The lower the signal to noise ratio, the worse the similarity to FDG PET is expected to be [54,62]. Also, the time between injection and acquisition start time partly determines correlation, as illustrated in Figure 3c, for instance for tau PET we see a clear drop in correlation to around 0.6 after 5 min. When computing the correlation, non-standardized time shifts between scans can bias the outcomes [69].

#### 3.2.4. Kinetic Modelling Parametric Images

R1, the relative tracer delivery parameter, reflects the ratio of the influx rate constant (K1) in target regions to that in a reference region and serves as a surrogate for relative cerebral blood flow. Most studies estimated R1 using simplified reference tissue models (SRTM1 or SRTM2), which are dependent on the choice of reference region. In most studies the whole cerebellum or cerebellar cortex (without superior layers) was used as the reference region, which is in line with recommendations for amyloid and tau PET [79,80] as these areas represent solely non-specific binding. As a consequence of the noisy early frames needed to compute kinetic parameters, resulting parametric images are prone to noise and need noise reduction [24,42]. When using partial volume corrections on the already noisy images, the variability is further increased [53,54].

When compared to FDG PET, R1 images showed correlations of 0.84 for [11C]-PiB, 0.78 for [18F]-FBP, 0.77 for [18F]-PI-2620, 0.85 for [18F]-THK5317, and 0.88 for [18F]-MK-6420 (against [15O]-H_2_O instead). The results lie in the same range as those for early phase scans and studies that directly compared both methods reported similar outcomes. Visually, R1 images are generally noisy and require noise reduction methods. Head-to-head comparisons between early phase and R1 images yielded high correlations (median (interquartile range (IQR)) r = 0.96 (0.89–0.98)), further supporting early phase PET as a robust measure of perfusion.

#### 3.2.5. Artificial Intelligence Techniques

A limited number of studies have explored AI approaches for deriving neurodegeneration measures from amyloid or tau PET. Three studies used machine learning (ML) for classification based on early phase PET, while two others applied deep learning (DL) to generate synthetic FDG PET images.

Matthews et al. [49] developed a ML model combining principal component analysis (PCA) and canonical variates analysis (CVA) to measure AD progression. They trained one model with early phase amyloid scans and one model with FDG PET. Ultimately, they found high correlations between classifier scores of both (r = 0.9). Similarly, Segovia et al. [66,67] developed a support vector machine (SVM) model and reported comparable performance using either FDG or early phase PET. Their model achieved ROC AUC scores of 83.7 and 84.3 when combining late-phase amyloid PET with FDG PET or early phase amyloid PET, respectively.

Recent DL methods showed promise in particular for image-to-image transformation. Choi et al. [31] employed a generative adversarial network (GAN) to generate pseudo-FDG PET scans from late-phase amyloid PET, achieving a structural similarity index measure (SSIM) of 0.77 and a peak signal-to-noise ratio (PSNR) of 32.4. Instead, Sanaat et al. [63] used a transformer neural network (TNN), a more advanced but computationally more expensive approach, that was trained on early phase amyloid scans using 5-fold cross validation. Their synthetic FDG images reached SSIM values of 0.90–0.94 and PSNR values of 30.2–30.6 for generated images against FDG, compared to initial values for early phase against FDG PET of 0.88–0.91 for SSIM and 25.4–25.9 for PSNR. Correlations increased from r = 0.78 to r = 0.82–0.84 for early phase and synthetic FDG images, respectively. Sanaat et al. [63] also evaluated clinically relevant uptake patterns and found a strong increase in similarity to FDG PET patterns using the synthetic images. For both healthy controls and AD patients, clinical similarity of uptake patterns increased from 1.96 to 2.63 (score 1: no similarity to 3: similar). However, the DL models were limited by a relatively small sample sizes (*n* < 170) and they were only trained and validated on patients along the AD continuum. No external validation sets were used. Bias could thus be introduced into the models; it remains unclear whether they accurately reproduce the nuanced regional patterns of other dementia pathologies, which is essential for differential diagnosis.

While AD-specific disease patterns are well validated for FDG PET, early phase images are not yet validated in the same way, but efforts are being made to address this [81]. Therefore, transforming early phase data into synthetic FDG-like images using AI may help bridge the subtle differences between the two modalities and help clinicians interpret these images.

### 3.3. Amyloid and Tau PET as Predictors of Both A and T Status

#### 3.3.1. General Characteristics

The characteristics of the seven studies exploring the potential of amyloid or tau PET to predict both A and T status in a single scan are summarized in Table 2. The research is rather novel and upcoming, as evidenced by the limited number of papers, all released after 2021. Therefore, one preprint and one conference abstract with novel approaches are also included. A similar number of studies investigated amyloid PET (*n* = 3) and tau PET (*n* = 4). As previously mentioned, tau PET becomes abnormal only after amyloid PET in the progression of AD and is more closely related to clinical symptoms. It is therefore hypothesized that tau PET could be used as an indicator of amyloid load. On the opposite end, the potential for amyloid PET to predict T status seems less plausible. A variety of radiotracers were used: [11C]-PiB, [18F]-FBP, [18F]-FTP, [18F]-PI-2620 and [18F]-MK-6420, and several ML and DL methods were explored for the prediction of A/T status or the generation of amyloid/tau PET scans.

#### 3.3.2. Tau PET to Predict A Status

Shcherbinin et al. [88] looked at a large sample (*n* = 1781) of patients with MCI or AD and evaluated the associations between tau and amyloid PET. They found that tau PET had a positive predictive value (PPV) of more than 93% in predicting amyloid positivity and a negative predictive value (NPV) of up to 77%. These results suggest that a positive tau PET scan is highly associated with a positive A status, consistent with the known AD neuropathological cascade. However, in the case of a negative tau PET scan the predictive value is significantly lower, indicating that the important distinction between A−/T− and A+/T− patients cannot be made using just a standard tau PET scan.

Three other studies used distinct methods to predict amyloid status from tau PET: using principal component analysis combined with machine learning, kinetic modelling approach and deep learning. They all found high predictive values and were also able to effectively predict A status from a negative tau scan. The first approach by Hammes et al. [83] was based on the difference in tau PET signatures for different neurodegenerative diseases. They used a scaled subprofile model principal component analysis (SSM/PCA) and found that the pattern expression strengths could predict amyloid status with a ROC AUC of 0.95. Additionally, they trained a SVM that achieved an accuracy of 98%. Secondly, Gnörich et al.* [82] (*Preprint) used the difference in kinetic binding properties of [18F]-PI-2620 to differentiate 3/4R-tauopathies from 4R-tauopathies, and as a result also indirectly the amyloid status. They used SRTM2 to derive the cortical tissue clearance (K2a) and found that it has a high predictive value of amyloid status: a PPV, NPV, and ROC AUC of 91.5%, 95.1%, and 0.99, respectively. It is important to note that at the moment of writing, this study is still a preprint and has not yet been peer reviewed. The third approach used deep learning to derive amyloid status. Ruwanpathirana et al. [87] developed a CNN (with 10-fold cross validation) to predict not just A status, but the centiloid score [89] from a tau PET input. They reported a predictive value R^2^ of 0.79 to predict the centiloid value.

#### 3.3.3. Amyloid PET to Predict T Status

**Figure 4:**
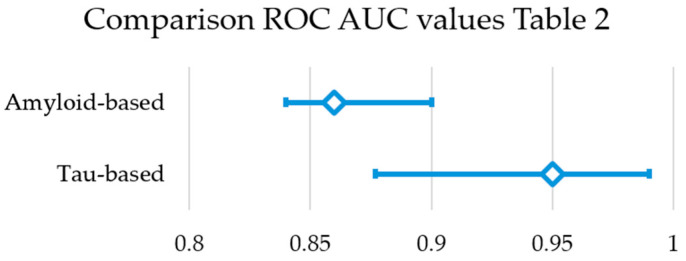
Comparison reported ROC AUC values of amyloid-based and tau-based studies as predictors of both A and T status (Table 2). The median and minimum-maximum range are displayed.

A small number of studies used amyloid PET to predict the T status. In comparison to tau PET for predicting A status, the ROC AUC values are slightly lower (0.84–0.9 as compared to 0.88–0.99), as visually represented in Figure 4. This suggests that tau PET is a stronger predictor for A status than vice versa. Nevertheless, the number of studies to compare is rather limited and differences could be attributed to patient demographics, study set-up, sample sizes, etc.

Raman et al. [86] used early phase amyloid PET and measured the standardized uptake value ratio (SUVR) within the hippocampus to predict tau positivity. They found a ROC AUC of 0.86. Early phase amyloid PET scan provides a perfusion-like image. As a consequence, this method is closer to using neurodegeneration (N) status properties to predict T status as opposed to amyloid related properties. Two other studies used DL techniques to generate synthetic tau PET images. Naseri et al. ** [85] (**Conference abstract) developed a conditional generative adversarial network (cGAN) for this purpose. They found image similarity metrics SSIM (structural similarity index measure) and PSNR (peak signal to noise ratio) of 0.917 and 27.04, respectively. They also reported a ROC AUC of 84%. However, only limited information on the methods and results is available as this study is a conference abstract. Lee et al. [84] developed a CNN that imputes tau PET images from amyloid PET, but also FDG PET (see next section). The generated image achieved a MS-SSIM (multiscale structural similarity index measure) higher than 0.94, validated using 5-fold cross validation. They found a ROC AUC higher than 0.9; however, when they compared it to a ROC analysis using the actual amyloid PET instead of the synthetic tau PET they found no significant difference.

### 3.4. Neurodegeneration Scans as Predictor A or T Status

#### 3.4.1. General Characteristics

The characteristics of the 12 studies exploring the potential of FDG PET or early phase surrogates to predict A or T status are summarized in Table 3. Again, this research is rather novel and upcoming, as evidenced by the limited number of papers, all released after 2021. In part, this is due to the rise in popularity of AI techniques, with five studies using machine learning and six studies using deep learning. The N status is a non-specific biomarker for AD, but by analyzing disease-specific PET signatures it is hypothesized that A and/or T status could be well inferred. The main advantage of this approach is that FDG PET is much more widely available compared to amyloid and tau PET imaging.

#### 3.4.2. FDG PET as Predictor A or T Status

Most studies (*n* = 8) explored FDG PET as a predictor of amyloid status. Median (IQR) ROC AUC values were 0.83 (0.80–0.87), which is slightly lower than studies from the previous section that used tau PET to predict amyloid status (ROC AUC = 0.95). Parmera et al. [95] investigated whether FDG PET patterns could predict amyloid deposition in a group of corticobasal syndrome patients. They found a PPV of 100% and a balanced accuracy of 88.5%. Several machine learning methods have been used to predict A status, including decision trees (DT), random forests (RF), gaussian naïve bayes (GNB), discriminant analysis (DA), and SVM. Results show a ROC AUC of 0.918–0.924 for the best performing models (SVM and GNB). Studies paid special attention to feature extraction methods using segmentation methods such as Statistical Parametric Mapping (SPM) or radiomics extraction methods. Most papers using deep learning techniques to determine A status reported ROC AUC values of 0.798–0.844, which were remarkably lower than those obtained from ML methods. A powerful advantage of deep learning is the ability to generate synthetic amyloid PET scans. Wang et al. [97] did an exploratory analysis, they found that predicted images had little overlap with true amyloid PET images. Zhou et al. [99] found similarity scores SSIM and NMSE (normalized mean squared error) of 0.764 and 14.58, respectively. These results could be attributed to the exploratory nature of the studies and the small sample sizes (*n* = 54 and *n* = 35) for DL training. However, they could also be an indication of the large differences in spatial patterns between amyloid and FDG PET.

Lee et al. [84] also used FDG PET to generate synthetic tau PET scans. They had also trained a CNN to generate tau PET from amyloid PET scans and compared this model to a version trained with FDG PET as an input. They used 5-fold cross validation and an external validation set. Regional correlations from FDG-based predictions (r > 0.8) were found to be higher than those for amyloid PET (r = 0.41–0.76). ROC AUC values were similar (>0.9) for both FDG and amyloid PET-based predictions; however, in this case the difference between AUC values using the true FDG scan and the FDG-based predictions was significantly different. This could be explained by the fact that while amyloid PET is inherently highly correlated with tau, for FDG the association is less straightforward (as it is non-specific for AD) and thus benefits more from a DL model that interprets its disease-specific patterns.

#### 3.4.3. Early Phase PET to Predict the Late-Phase PET Status

Three studies explored early phase amyloid PET as a predictor of A status. The clinical value of this approach is rather limited, since late-phase scans are considered the standard and saving 60 min does not justify the reduced accuracy from an early phase prediction. However, this is interesting when looking at it from the perspective of the early phase being an FDG surrogate. As simultaneous pairs of early phase and late-phase images are easier to obtain, it provides new data to explore the associations between neurodegeneration and amyloid PET. Similar results were found between FDG-predicted A status (ROC AUC = 0.798–0.924) and early phase-predicted A status (ROC AUC = 0.779–0.83). Komori et al. [93] generated late-phase amyloid PET from early phase scans using a CNN and found a SSIM of 0.45.

## 4. Discussion

In this review we provided an overview of the literature of how PET imaging (amyloid, tau, and FDG PET) can determine the A, T, and N status. For each possible prediction scheme (amyloid PET to T/N, tau PET to A/N, and FDG PET to A/T), at least one publication was found. Using a single PET scan to predict multiple biomarkers reliably, would allow for a significantly reduced radiation dose, tracer costs, and time commitment for the patients and would also be cost-effective for the healthcare system in light of the new disease modifying drugs for AD.

The most investigated concept was early phase amyloid or tau PET as a surrogate for neurodegeneration. This also seems the most plausible for clinical practice, as it is relatively easy to obtain as part of a dual-phase protocol and does not require advanced processing techniques. Across multiple radiotracers, early phase signals have consistently shown strong correlations with FDG PET. Furthermore, the results were shown to be independent of amyloid or tau status, even in non-AD pathologies. An important parameter is the window size. Some ultrashort (1 min) scans have shown very high correlations, but these protocols might be subject to more noisy images. As noise is reduced with new PET scanners gaining higher resolution and sensitivity, this obstacle will probably become less important. Parametric R1 maps of relative perfusion have also been shown to highly correlate with the N status, which were not significantly different than early phase PET correlations. Theoretically, R1 images provide a more specific estimate of tracer delivery and are more reproducible than (short) early phase scans as they do not depend on the choice of the early phase time window. However, they require longer dynamic scan times and specialized modelling software and therefore are more difficult to implement in clinical practice. Kinetic modelling is also more difficult to standardize across centra, as the different software have substantial differences in their processing pipelines (such as for motion correction and segmentation of reference regions). Therefore, early phase scans appear to be the preferred option for widespread clinical application. One study [63] explored a deep learning network for generation of synthetic FDG PET scans from early phase amyloid PET scans and reported promising results. With deep learning becoming increasingly relevant in medical imaging, it should be explored further for other tracers, including tau PET, and across more varied patient populations. Moreover, AI might help clinicians to interpret early phase images as subtle variations between early phase and FDG PET scans are observed, mainly in the subcortical and occipital regions. However, obtaining large, high-quality datasets needed to train and validate a robust DL model is still a major challenge.

Amyloid and tau PET are highly associated though they reflect pathological changes at different time points. It is generally accepted that amyloid PET becomes abnormal first and probably years before symptom onset, while pathological tau PET occurs later and closer to first expressions of symptoms. Based on the hypothesis that tau deposition is a downstream effect of amyloid accumulation, it was expected that tau PET could predict A status, whereas the reverse, i.e., predicting T status from amyloid PET would be more difficult. Research on this subject remains limited, with only four publications about tau PET to amyloid prediction and three publications about amyloid PET to tau prediction. Despite the substantial visual and diagnostic differences between both modalities, these publications reported surprisingly good results for both cases. Again, deep learning methods were explored for both the prediction of A/T status and the generation of synthetic amyloid/tau PET images and showed promising results. The strength of AI lies in its ability to interpret large amounts of data and find complex relations. In this case, AI has been shown to help predict multiple biomarkers, but it can also improve our knowledge of biomarker interactions. One preprint study [82] explored a very interesting way of predicting amyloid load from a tau PET scan using cortical tissue clearance (K2a) obtained by kinetic modelling and reported a remarkable AUC of 0.99. Overall, these promising results underscore significant potential in this area, but the very limited number of publications shows a clear research gap that warrants further research.

Lastly, while FDG PET is not a specific biomarker for AD, it is reasonable to assume that characteristic PET patterns could be used to predict or differentiate amyloid or tau status. The main advantage of FDG PET as a predictor, is its wide availability as it is already a standard of clinical practice. However, the association between FDG PET and A or T status is more difficult to obtain and thus is expected to provide worse results compared to the other prediction schemes. Nevertheless, several studies have reported relatively high outcome measures, especially for ML methods. DL methods showed more difficulty in generating a synthetic amyloid PET image compared to previous tau PET-based methods. Only one study explored FDG PET to generate tau PET and found slightly better results compared to amyloid PET to tau PET synthesis. In line with this study, others explored early phase amyloid scans to predict A status, and although this is clinically not that useful, it provides similar insights as if using FDG PET instead. Overall, research in this area remains limited and future research might provide valuable information on the potential of deriving A and T status from FDG patterns.

PET imaging is associated with radiation exposure, with an estimated effective dose of approximately 1.9 mSv for a standard [18F]-FDG brain PET scan (100 MBq) [100], and between 4 and 6 mSv for amyloid and tau PET (150 MBq) [79,80,101,102,103]. However, recent trends can lead to major dose reduction. Firstly, scanners with a longer axial field of view (FOV) and higher detector sensitivities (e.g., BGO crystal systems) allow for shorter acquisition times or lower dose scans. Secondly, dose reduction can be achieved by the improvements in time-of-flight (TOF) performance, with the latest systems achieving timing resolutions below 200 ps. And thirdly, DL techniques can reduce noise associated with low dose acquisitions in the reconstructed PET image. Some studies have already explored (ultra) low dose imaging for AD related brain PET scans [104,105,106].

While the focus of the review is on MCI and AD patients, good results were found across all disease stages (CU, MCI, and dementia). For early phase perfusion as a surrogate for N, no significant differences were found between diagnostic groups. However, it can be hypothesized that more severe hypometabolism patterns seen in advanced AD patients increase the correlation with A and T status simply because of the larger range of values. Moreover, in Shcherbinin et al. [88] it was observed that a positive tau scan predicts amyloid positivity simply because of probability, as A+T+ patients are much more common than A−T+ patients.

Several concerns limit the current clinical feasibility of a single-tracer PET. Especially since tau tracer availability remains limited, with currently only one European Medicines Agency (EMA) approved tau tracer. Moreover, reimbursement for tau and amyloid PET if any, is still limited and strictly regulated in most European countries complicating wide clinical availability. In contrast, FDG PET is widely available and reimbursed in many settings and would currently be the most practically feasible as a single-tracer PET scan. However, as illustrated by the NIA-AA concept N is a specific marker and using only FDG PET scans A and T status show lower median (IQR) ROC AUC values of 0.83 (0.80–0.87).

This review has some limitations. The number of publications for certain subjects remains limited and the wide variety of methods reduces the robustness of direct comparisons and interpretations (such as the large variety of analyses performed, choices of ROIs, scan protocols, demographics, criteria for A or T positivity, etc.). To address this, we focused on identifying common patterns and overarching trends rather than making direct quantitative comparisons between studies. A large portion of studies had ‘high’ or ‘unclear’ patient selection bias and ‘high’ or ‘unclear’ flow and timing bias. When the aim of the study is not considered during the enrolment of patients, such as in retrospective studies, population bias can be introduced. This often meant that it was also not ensured that images were processed in a similar way or taken within a reasonable time frame from each other, thus also introducing flow and timing bias.

To summarize, amyloid, tau, and FDG PET are closely correlated and are predictive of each other to differing degrees. Early phase amyloid or tau PET demonstrated feasibility as a surrogate measure of neurodegeneration, with high similarities to FDG PET. Nevertheless, the heterogeneity of the underlying studies, such as the variability in study design, time windows, tracer types, sample sizes, and disease stages, introduce uncertainty in whether early phase amyloid or tau PET could reliably replace FDG PET. This highlights the need for standardization of perfusion disease patterns, as has been established for FDG PET. Future efforts should be made to overcome remaining challenges [81]: (1) the need for standardized acquisition protocols (such as a standardized time window, we suggest from 1 to 2 min postinjection), (2) availability of normal perfusion datasets and templates, (3) dedicated and clinically approved software for (semi-)quantification, and (4) validation of clinical value (especially in MCI patients) in prospective clinical trials. Among the available modalities, tau PET then seems the strongest candidate for a single tracer examination to determine all three biomarkers. This is supported by its better performance in predicting the A status than amyloid PET was for predicting T status, as suggested by preliminary studies, and aligns with by the concept of tau expression occurring only after amyloid deposition in AD. However, as research is still very limited in this field, future research should focus on amyloid-tau prediction schemes. Besides the potential to reduce the need for multiple PET scans, drastically reducing radiation exposure (reduced by a factor of (3) and increasing cost-effectiveness for both patient and healthcare system, this research could provide valuable insights into the interactions between amyloid, tau, and neurodegeneration and may help to understand the pathophysiology of AD and other dementia syndromes.

## Figures and Tables

**Table 1:** Characteristics of 58 studies that use amyloid or tau PET as predictors of N status. A smaller selection of outcome measures, VOI-based correlation (Pearson r or Spearman ρ) and ROC AUC scores, is reported for each study to ensure readability. Quantitative measures are reported as the mean or a range of values. Values that are preceded by a tilde ‘~’ are estimated based on, e.g., graphs, if not explicitly reported. Area under the curve (AUC) values are reported for dementia (usually AD) versus HC. ‘/’ means the study did not report these specific outcome measures. A more extensive table listing additional outcome measures can be found in Table S1 of the Supplementary Material.

Ref.	Author, Year	PET Radiotracer	Methodology vs. Comparator	Sample Size (*n*)	Outcome Measures
[21]	Albano et al., 2022	[18F]-FBP	1–6 min vs. FDG	12	r = 0.89
[22]	Asghar et al., 2019	[18F]-FBP	2–5 min vs. FDG	28	r = 0.79
[23]	Aye et al., 2024	[18F]-FBB	0–10 min vs. ASL MRI	115	r = 0.15–0.49 and ROC AUC = 0.83
[24]	Beyer et al., 2020	[18F]-PI-2620	0.5–2.5 min, R1 vs. FDG	26	r = 0.76 (0.5–2.5 min), r = 0.77 (R1)
[25]	Bilgel et al., 2020	[11C]-PiB	0.75–2.5 min, R1 vs. H_2_O	149	r = 0.79 (0.75–2.5 min), r = 0.76 (R1)
[26]	Boccalini et al., 2023	[18F]-FBP, [18F]-FMM	0–5 min vs. FDG, 0–10 min vs. FDG	166	r = 0.79 (FBP), r = 0.81 (FMM) and ROC AUC = 0.80–0.89
[27]	Boccalini et al., 2025	[18F]-FTP	0–10 min vs. FDG	58	r = 0.84 and ROC AUC = 0.60
[28]	Bunai et al., 2019	[11C]-PiB	1–8 min vs. FDG	95	r = 0.63–0.94
[29]	Carneiro et al., 2022	[11C]-PiB	0–10 min vs. FDG	90	r = ~0.70–0.95
[30]	Chen et al., 2015	[11C]-PiB	R1 vs. H_2_O	19	ρ = ~0.80–0.90
[31]	Choi et al., 2023	[18F]-FBB	DL (90–110 min) vs. FDG	110	/
[32]	Daerr et al., 2017	[18F]-FBB	0–5 min, 0–10 min vs. FDG	33	r = 0.86
[33]	Dghoughi et al., 2019	[18F]-FMM	0–1 min vs. FDG	19	r = 0.76
[34]	Fettahoglu et al., 2024	[18F]-FBB	0–2 min vs. H_2_O	20	r = 0.90
[35]	Florek et al., 2018	[18F]-FBB	0–10 min	112	/
[36]	Forsberg et al., 2012	[11C]-PiB	0–6 min vs. FDG	64	r = ~0.39–0.74
[37]	Fu J. et al., 2025	[18F]-MK-6420	0–3 min, R1 vs. H_2_O	17	r = 0.84, r = 0.88
[38]	Fu L. et al., 2014	[11C]-PiB	1.33–8 min vs. FDG	40	r = 0.87
[39]	Gómez-Grande et al., 2023	[18F]-FBP, [18F]-FMM	0–1 min vs. FDG, 0–1 min vs. FDG	17	r = 0.92
[40]	Guehl et al., 2023	[18F]-MK-6420, [11C]-PiB	R1 (MK-6420) vs. R1 (PiB)	49	r = 0.95
[41]	Hammes et al., 2017	[18F]-FTP	1–6 min vs. FDG	20	r = ~0.82–0.95
[42]	Hsiao et al., 2012	[18F]-FBP	0–2 min, 1–6 min, R1 vs. FDG	14	r = 0.78 (0–2 min), r = 0.87 (1–6 min), r = 0.78 (R1)
[43]	Jeong et al., 2019	[18F]-FPN	0–10 min vs. FDG	33	r = 0.83
[44]	Joseph-Mathurin et al., 2018	[11C]-PiB	1–9 min, R1 vs. H_2_O	110	/
[45]	Kwon et al., 2021	[18F]-FBB	0–10 min vs. ECD SPECT	27	r = 0.90 and ROC AUC = 0.91
[46]	Leuzy et al., 2018	[18F]-THK5317	0–3 min, R1 vs. FDG	16	r = 0.83 (0–3 min), r = 0.85 (R1)
[47]	Lin et al., 2016	[18F]-FBP	1–6 min	82	/
[48]	Lojo-Ramírez et al., 2025	[18F]-FBB	0–5 min vs. FDG	103	ρ = 0.88 and ROC AUC = 0.86
[49]	Matthews et al., 2022	[18F]-FBP	ML (0–6 min) vs. FDG	111	/
[50]	Meyer et al., 2011	[11C]-PiB	R1 vs. FDG	22	r = 0.79
[51]	Myoraku et al., 2022	[18F]-FBP, [18F]-FBB	0.75–6 min vs. FDG, 0.75–6 min vs. FDG	100	r = 0.74
[52]	Oliveira et al., 2018	[11C]-PiB	0–6 min, 1–8 min, R1 vs. FDG	52	/
[53]	Ottoy et al., 2019	[18F]-FBP	0–2 min, R1 vs. H_2_O	39	r = 0.70–0.94 (0–2 min), r = 0.65–0.92 (R1) and ROC AUC = 0.87–0.95 (0–2 min), 0.86–0.95 (R1)
[54]	Peretti et al., 2019	[11C]-PiB	20–130 s, R1 vs. FDG	30	r = 0.76 (20–130 s), r = 0.85 (R1)
[55]	Peretti et al., 2019	[11C]-PiB	20–130 s, 1–8 min, R1 vs. FDG	52	ROC AUC = 0.94 (20–130 s), 0.89 (1–8 min), 0.92 (R1)
[56]	Peretti et al., 2021	[11C]-PiB	R1 vs. FDG	79	ROC AUC = 0.81
[57]	Peretti et al., 2022	[11C]-PiB	20–130 s, 1–8 min, R1 vs. FDG	52	r = 0.59 (20–130 s), r = 0.49 (1–8 min), r = 0.79 (R1) and ROC AUC = 0.69 (20–130 s), 0.85 (1–8 min), 0.83 (R1)
[58]	Ponto et al., 2019	[11C]-PiB	3.5–4 min, 0–6 min, R1 vs. H_2_O	24	r = 0.61 (3.5–4 min), r = 0.52 (0–6 min), r = 0.62 (R1)
[59]	Ribaldi et al., 2025	[18F]-FBP, [18F]-FMM	0–5 min vs. ASL MRI, 0–10 min vs. ASL MRI	46	/
[60]	Rodriguez-Vieitez et al., 2016	[11C]-PiB	1–4 min vs. FDG	41	r = 0.61 and ROC AUC = 0.84–0.90
[61]	Rodriguez-Vieitez et al., 2017	[18F]-THK5317, [11C]-PiB	0–3 min, R1 vs. FDG, 1–8 min, R1 vs. FDG	20	r = 0.86 (THK), r = 0.88 (PiB), r = 0.86 (R1 THK), r = 0.90 (R1 PiB) and ROC AUC = 0.82 (THK), 0.78 (PiB), 0.84 (R1 THK), 0.79 (R1 PiB)
[62]	Rostomian et al., 2011	[11C]-PiB	1–8 min vs. FDG	83	r = 0.91
[63]	Sanaat et al., 2024	[18F]-FBP, [18F]-FMM	DL (0–5 min) vs. FDG, DL (0–10 min) vs. FDG	166	r = 0.82 (FBP), r = 0.85 (FMM)
[64]	Schmitt et al., 2021	[18F]-FMM	0–10 min vs. FDG	20	r = 0.86
[65]	Segovia et al., 2018	[18F]-FBB	0–10 min vs. FDG	47	r = ~0.5
[66]	Segovia et al., 2018	[18F]-FBB	ML vs. FDG	47	/
[67]	Segovia et al., 2020	[18F]-FBB	ML (0–20 min) vs. FDG	43	ROC AUC > 0.8
[68]	Seiffert et al., 2020	[18F]-FBP	0–10 min vs. FDG	19	r = 0.72
[69]	Seiffert et al., 2021	[18F]-FBP, [18F]-FBB, [18F]-FMM	0–1 min vs. FDG, 0–1 min vs. FDG, 0–1 min vs. FDG	60	r = 0.86 (FBP), r = 0.77 (FBB), r = 0.78 (FMM)
[70]	Son et al., 2020	[18F]-FBB	0–5 min vs. FDG	40	r = ~0.77
[71]	Tiepolt et al., 2016	[11C]-PiB, [18F]-FBB	1–9 min vs. FDG, 1–9 min vs. FDG	22	r = 0.73 (PiB), r = 0.81 (FBB)
[72]	Tiepolt et al., 2019	[11C]-PiB	1–9 min	31	/
[73]	Tuncel et al., 2023	[18F]-FBP, [18F]-FTP	R1 (FBP) vs. R1 (FTP)	50	r = 0.89–0.93
[74]	Vanhoutte et al., 2021	[18F]-FBP	0–4 min vs. FDG	191	/
[75]	Völter et al., 2023	[18F]-PI-2620, [18F]-FMM	0.5–2.5 min (PI-2620) vs. 0–10 min (FMM)	64	r = 0.82
[76]	Völter et al., 2025	[18F]-FBB, [18F]-FMM	0–10 min (FBB), 0–10 min (FMM)	82	/
[77]	Wolters et al., 2020	[18F]-FTP	R1 vs. FDG	133	AUC = 0.94
[78]	Yoon et al., 2021	[18F]-FBB	0–10 min vs. R1	60	r = 0.75–0.91

Abbreviations: ROC AUC = Receiver Operating Characteristic Area Under the Curve; ML = machine learning; DL = deep learning; VOI = volume of interest; R1 = relative delivery rate; ASL MRI = arterial spin labelling magnetic resonance imaging.

**Table 2:** Characteristics of 7 studies that used amyloid or tau PET as predictors of both A and T status.

Ref.	Author, Year	PET Radiotracer	Methodology (Specified Model)	Sample Size (*n*)	Outcome Measures
[82]	Gnörich et al., 2025 *	[18F]-PI-2620	K2a using kinetic modelling (SRTM2)	146	prediction of A status ROC AUC = 0.99, PPV = 0.915, NPV = 0.951
[83]	Hammes et al., 2021	[18F]-FTP	SSM/PCA + ML (SVM)	54	prediction of A status ROC AUC = 0.95, SS = 0.94, SP = 0.83
[84]	Lee et al., 2024	[11C]-PiB	DL (CNN)	1480	generation of tau PET correlation r = 0.41–0.76, ROC AUC > 0.9
[85]	Naseri et al., 2023 **	[18F]-FBP	DL (cGAN)	475	generation of tau PET ROC AUC = 0.84, SSIM = 0.917
[86]	Raman et al., 2022	[18F]-FBP	early phase	410	prediction of T status ROC AUC = 0.86, SS = 0.71, SP = 0.93
[87]	Ruwanpathirana et al., 2022	[18F]-MK6240	DL (CNN)	134	prediction of centiloid score RMSE = 29.93, R^2^ = 0.79
[88]	Shcherbinin et al., 2023	[18F]-FTP	late-phase	1781	prediction of A status PPV ≥ 93%, NPV = 60–77%, ROC AUC = 0.88

* Preprint ** Conference abstract. Abbreviations: ROC AUC = Receiver Operating Characteristic Area Under the Curve; PPV = positive predictive value; NPV = negative predictive value; SS = sensitivity; SP = specificity; SSIM = structural similarity index; RMSE = root mean square error; SRTM2 = simplified reference tissue model 2; SSM/PCA = scaled subprofile model principal component analysis; ML = machine learning; SVM = support vector machine; DL = deep learning; CNN = convolutional neural network; cGAN = conditional generative adversarial network.

**Table 3:** Characteristics of 12 studies that use FDG PET or surrogates as predictors of both A and T status.

Ref.	Author, Year	PET Radiotracer	Methodology (Specified Model)	Sample Size (*n*)	Outcome Measures
[90]	Alongi et al., 2022	[18F]-FDG	ML (DA)	43	prediction of A status SS = 84.92%, SP = 75.13%, PR = 73.75% and ACC = 79.56%
[91]	Ardakani et al., 2025	[18F]-FDG	DL (CNN)	286	prediction of A status ROC AUC = 0.815–0.844 and F1 Score = 0.770–0.809
[92]	Choi et al., 2025	eFBB	ML (DT, RF, GB, and more)	176	prediction of A status ROC AUC = 0.83 and F1 Score = 0.80
[15]	Kim et al., 2021	[18F]-FDG	DL (CNN)	1533	prediction of A status ROC AUC = 0.798–0.811 and F1 Score = 0.709–0.712
[93]	Komori et al., 2022	ePiB	DL (CNN)	253	generation of delayed PET image intra-reader agreement κ = 0.59–0.60 and inter-reader agreement κ = 0.79 SSIM = 0.45 and PSNR = 21.8
[84]	Lee et al., 2024	[18F]-FDG	DL (CNN)	1480	generation of tau PET correlation r > 0.8, ROC AUC > 0.9
[94]	Park et al., 2025 *	eFMM	ML (LR, DA)	454	prediction of A status ROC AUC = 0.779–0.791
[95]	Parmera et al., 2021	[18F]-FDG	\	45	prediction of A status SS = 76.92%, SP = 100%, PPV = 100%, ACC = 88.5%
[96]	Rasi et al., 2024	[18F]-FDG	ML (RF, GNB, and more)	301	prediction of A status ROC AUC = 0.924
[97]	Wang et al., 2021	[18F]-FDG	DL (CNN)	54	generation of amyloid PET exploratory, visual analysis
[98]	Yamada et al., 2025	[18F]-FDG	ML (SVM)	194	prediction of A status ROC AUC = 0.918
[99]	Zhou et al., 2021	[18F]-FDG	DL (GAN)	35	generation of amyloid PET SSIM = 0.764 and NMSE = 14.58

* No full text could be accessed. Abbreviations: ROC AUC = Receiver Operating Characteristic Area Under the Curve; PPV = positive predictive value; SS = sensitivity; SP = specificity; PR = precision; ACC = accuracy; SSIM = structural similarity index; NMSE = normalized mean square error; PSNR = peak signal to noise ratio; ML = machine learning; DA = discriminant analysis; SVM = support vector machine; DT = decision tree; RF = random forest; GB = gradient boosting; LR = logistic regression; DL = deep learning; CNN = convolutional neural network; GAN = generative adversarial network.

## Data Availability

No new data were created or analyzed in this study. Data sharing is not applicable to this article.

## Supplementary Materials

The following supporting information can be downloaded at: https://www.mdpi.com/article/10.3390/brainsci15121271/s1, Detailed search query; Table S1: Characteristics of 58 studies that use amyloid or tau PET as predictors of N status.

## Appendix A

Quality assessment was performed for all included studies using the Quality Assessment of Diagnostic Accuracy Studies-2 (QUADAS-2) tool. Each study’s risk of bias was rated low, high or unclear for patient selection, index test, reference standard, and flow and timing. Concerns regarding applicability to the review question (how PET imaging can determine multiple biomarkers for AD) was also rated low, high or unclear for patient selection, index test, and reference standard. Standard signalling questions were used to help reach judgements [20]. Results are represented in tabular (Table A1) and graphical (Figure A1) form below.

**Table A1:** Assessment of bias and applicability concerns of the 76 included studies using the Quality Assessment of Diagnostic Accuracy Studies-2 (QUADAS-2) tool.

Ref.	Author, Year	Risk of Bias				Applicability Concerns		
		Patient Selection	Index Test	Reference Standard	Flow and Timing	Patient Selection	Index Test	Reference Standard
[21]	Albano et al., 2022	Low	Low	Low	Low	Low	Low	Low
[22]	Asghar et al., 2019	High	Low	Low	High	Low	Low	Low
[23]	Aye et al., 2024	High	Low	Unclear	Low	Low	Low	Unclear
[24]	Beyer et al., 2020	Low	High	Low	High	Low	Low	Low
[25]	Bilgel et al., 2020	High	Low	Low	Low	Unclear	Low	Low
[26]	Boccalini et al., 2023	Low	Low	Low	High	Low	Low	Low
[27]	Boccalini et al., 2025	Low	Low	Low	High	Low	Low	Low
[28]	Bunai et al., 2019	Low	Low	Low	Low	Low	Low	Low
[29]	Carneiro et al., 2022	Low	Low	Low	Low	Low	Low	Low
[30]	Chen et al., 2015	Low	Low	Low	Unclear	Low	Low	Low
[31]	Choi et al., 2023	High	Unclear	Unclear	Unclear	Low	Unclear	Low
[32]	Daerr et al., 2017	Low	Low	Low	High	Low	Low	Low
[33]	Dghoughi et al., 2019	High	Low	Low	Unclear	Low	Low	Low
[34]	Fettahoglu et al., 2024	Low	Low	Low	Unclear	Low	Low	Low
[35]	Florek et al., 2018	High	Low	High	Unclear	Low	Low	High
[36]	Forsberg et al., 2012	Low	Low	Low	Unclear	Low	Low	Low
[37]	Fu J. et al., 2025	Low	Low	Low	High	Low	Low	Low
[38]	Fu L. et al., 2014	Low	Low	Low	Low	Low	Low	Low
[39]	Gómez-Grande et al., 2023	High	Low	Low	Unclear	Low	Low	Low
[40]	Guehl et al., 2023	Low	Low	High	Unclear	Low	Low	High
[41]	Hammes et al., 2017	Low	Low	Low	Low	Low	Low	Low
[42]	Hsiao et al., 2012	Unclear	Low	Low	Unclear	Low	Low	Low
[43]	Jeong et al., 2019	High	Low	Low	Low	Low	Low	Low
[44]	Joseph-Mathurin et al., 2018	Low	Low	Low	High	Low	Low	Low
[45]	Kwon et al., 2021	High	Low	Unclear	Low	Low	Low	Low
[46]	Leuzy et al., 2018	Low	Low	Low	Unclear	Low	Low	Low
[47]	Lin et al., 2016	Low	Low	High	Unclear	Low	Low	High
[48]	Lojo-Ramírez et al., 2025	High	Low	Low	High	Low	Low	Low
[49]	Matthews et al., 2022	High	Low	Low	High	Low	Low	Low
[50]	Meyer et al., 2011	Low	Low	Low	Low	Low	Low	Low
[51]	Myoraku et al., 2022	High	Low	Low	High	Low	Low	Low
[52]	Oliveira et al., 2018	Low	Low	Low	Low	Low	Low	Low
[53]	Ottoy et al., 2019	Low	Low	Low	High	Low	Low	Low
[54]	Peretti et al., 2019	Unclear	Low	Low	Low	Low	Low	Low
[55]	Peretti et al., 2019	Unclear	Low	Low	Low	Low	Low	Low
[56]	Peretti et al., 2021	Unclear	Low	Low	Low	Low	Low	Low
[57]	Peretti et al., 2022	Unclear	Low	Low	Low	Low	Low	Low
[58]	Ponto et al., 2019	Low	Low	Low	Unclear	Low	Low	Low
[59]	Ribaldi et al., 2025	High	Low	Unclear	Unclear	Low	Low	Unclear
[60]	Rodriguez-Vieitez et al., 2016	Unclear	Low	Low	Unclear	Low	Low	Low
[61]	Rodriguez-Vieitez et al., 2017	Unclear	Low	Low	Unclear	Low	Low	Low
[62]	Rostomian et al., 2011	Low	Low	Low	Unclear	Low	Low	Low
[63]	Sanaat et al., 2024	Low	Low	Low	High	Low	Low	Low
[64]	Schmitt et al., 2021	Low	Low	Low	High	Low	Low	Low
[65]	Segovia et al., 2018	Low	Low	Low	Low	Low	Low	Low
[66]	Segovia et al., 2018	Low	Unclear	Low	Unclear	Low	Unclear	Low
[67]	Segovia et al., 2020	Low	Low	Low	Low	Low	Low	Low
[69]	Seiffert et al., 2021	High	Low	Low	Unclear	Low	Low	Low
[68]	Seiffert et al., 2020	High	Low	Low	Unclear	Low	Low	Low
[70]	Son et al., 2020	Low	Low	Low	Low	Low	Low	Low
[71]	Tiepolt et al., 2016	High	Low	Low	Unclear	Low	Low	Low
[72]	Tiepolt et al., 2019	High	Low	High	Unclear	Low	Low	High
[73]	Tuncel et al., 2023	Low	Low	High	Low	Unclear	Low	High
[74]	Vanhoutte et al., 2021	High	Low	Low	Low	Low	Low	Low
[75]	Völter et al., 2023	Low	Low	High	Low	Low	Low	High
[76]	Völter et al., 2025	High	Low	High	Low	Low	Low	High
[77]	Wolters et al., 2020	High	Low	Low	High	Low	Low	Low
[78]	Yoon et al., 2021	High	Low	High	Unclear	Low	Low	High
[82]	Gnörich et al., 2025 *	Low	Low	Low	Unclear	Low	Low	Low
[83]	Hammes et al., 2021	Low	Low	Low	Unclear	Low	Low	Low
[84]	Lee et al., 2024	High	Low	Low	Unclear	Low	Low	Low
[85]	Naseri et al., 2023 **	High	Unclear	Unclear	Unclear	Low	Unclear	Unclear
[86]	Raman et al., 2022	Unclear	Low	Low	High	Low	Low	Low
[87]	Ruwanpathirana et al., 2022	Unclear	Low	Low	Unclear	Low	Low	Low
[88]	Shcherbinin et al., 2023	High	Low	Low	Unclear	Low	Low	Low
[90]	Alongi et al., 2022	Low	Low	Low	High	Low	Low	Low
[91]	Ardakani et al., 2025	Unclear	Low	Low	Low	Low	Low	Low
[92]	Choi et al., 2025	High	Low	Low	Unclear	Low	Low	Low
[15]	Kim et al., 2021	High	Low	Low	Unclear	Low	Low	Low
[93]	Komori et al., 2022	High	Low	Low	Low	Low	Low	Low
[94]	Park et al., 2025 ***	Unclear	Unclear	Unclear	Unclear	Unclear	Unclear	Unclear
[95]	Parmera et al., 2021	Low	Low	Low	High	Low	Low	Low
[96]	Rasi et al., 2024	High	Low	Low	High	Low	Low	Low
[97]	Wang et al., 2021	Unclear	Low	Low	Unclear	Low	Low	Low
[98]	Yamada et al., 2025	High	Low	Low	Unclear	Low	Low	Low
[99]	Zhou et al., 2021	Unclear	Low	Low	Unclear	Low	Low	Low

* Preprint ** Conference abstract *** No full text could be accessed.
