# Supplementary material for: Correlation and Interchangeability of Amyloid, Tau, and Glucose Metabolism PET in Mild Cognitive Impairment and Alzheimer: A Review
Source: Brain Sci. 2025 Nov 26;15(12):1271. doi: 10.3390/brainsci15121271 (PMC12730394; doi:10.3390/brainsci15121271)
Supplement: Supplementary file 1 [file brainsci-15-01271-s001.zip › brainsci-3985069-supplementary.pdf]

Supplementary Materials

1. Detailed search query

Search: (surrogate OR predict OR correlate) AND (tau PET OR amyloid PET) AND (Alzheimer* OR dementia) NOT (plasma) NOT (mouse)

Detailed search query generated by PubMed: ((("surrogate"[All Fields] OR "surrogates"[All Fields] OR ("predict"[All Fields] OR "predictabilities"[All Fields] OR "predictability"[All Fields] OR "predictable"[All Fields] OR "predictably"[All Fields] OR "predicted"[All Fields] OR "predicting"[All Fields] OR "prediction"[All Fields] OR "predictions"[All Fields] OR "predictive"[All Fields] OR "predictively"[All Fields] OR "predictiveness"[All Fields] OR "predictives"[All Fields] OR "predictivities"[All Fields] OR "predictivity"[All Fields] OR "predicts"[All Fields]) OR ("correlate"[All Fields] OR "correlated"[All Fields] OR "correlates"[All Fields] OR "correlating"[All Fields] OR "correlation"[All Fields] OR "correlation s"[All Fields] OR "correlations"[All Fields] OR "correlative"[All Fields] OR "correlatives"[All Fields])) AND ((("transl androl urol"[Journal] OR "tau"[All Fields]) AND "PET"[All Fields]) OR (("amyloid"[Supplementary Concept] OR "amyloid"[All Fields] OR "amyloids"[All Fields] OR "amyloid"[MeSH Terms] OR "amyloidal"[All Fields] OR "amyloidic"[All Fields] OR "app protein human"[Supplementary Concept] OR "app protein human"[All Fields]) AND "PET"[All Fields])) AND ("alzheimer*"[All Fields] OR ("dementia"[MeSH Terms] OR "dementia"[All Fields] OR "dementias"[All Fields] OR "dementia s"[All Fields]))) NOT ("plasma"[MeSH Terms] OR "plasma"[All Fields] OR "plasmas"[All Fields] OR "plasma s"[All Fields])) NOT ("mice"[MeSH Terms] OR "mice"[All Fields] OR "mouse"[All Fields] OR "mouse s"[All Fields] OR "mouses"[All Fields])

2. Extended summary studies in group 1

Table S1: Characteristics of 58 studies that use amyloid or tau PET as predictors of N status. A selection of outcome measures is reported for each study to ensure readability. Quantitative measures are reported as the mean or a range of values. Values that are preceded by a tilde '~' are estimated based on e.g. graphs, if not explicitly reported. Area under the curve (AUC) values are reported for dementia (usually AD) versus HC.

Ref.	Author, Year	PET radiotracer	Methodology	Optimal time window	Comparator	Sample size (n)	Outcome measures
[21]	Albano et al., 2022	[18F]-FBP	ePET	1-6 min	[18F]-FDG	12	VOI-based correlation: r=0.89; intra-reader agreement: κ=0.83
[22]	Asghar et al., 2019	[18F]-FBP	ePET	2-5 min	[18F]-FDG	28	VOI-based correlation: r=0.79; voxel-wise analysis
[23]	Aye et al., 2024	[18F]-FBB	ePET	0-10 min	ASL MRI	115	VOI-based correlation: r=0.15-0.49; voxel-wise analysis; ROC AUC=0.83
[24]	Beyer et al., 2020	[18F]-PI-2620	ePET, R1 (SRTM2)	0.5-2.5 min	[18F]-FDG	26	VOI-based correlation: r=0.76, r=0.77; optimal timeframe analysis
[25]	Bilgel et al., 2020	[11C]-PiB	ePET, R1 (SRTM)	0.75-2.5 min	[15O]-H2O	149	VOI-based correlation: r=0.79, r=0.76; meta-ROI ICC=0.81, ICC=0.86
[26]	Boccalini et al., 2023	[18F]-FBP, [18F]-FMM	ePET	0-5 min, 0-10 min	[18F]-FDG	166	VOI-based correlation: r=0.79, r=0.81; voxel-wise analysis; ROC AUC=0.80-0.89; correlation with MMSE=0.41-0.48
[27]	Boccalini et al., 2025	[18F]-FTP	ePET	0-10 min	[18F]-FDG + eFBP/eFMM	58	VOI-based correlation: r=0.84; voxel-wise analysis; ROC AUC=0.60; correlation with MMSE=0.44; also compared to early phase amyloid
[28]	Bunai et al., 2019	[11C]-PiB	ePET	1-8 min	[18F]-FDG	95	VOI-based correlation: r=0.63-0.94; SS=0.82, SP=0.97
[29]	Carneiro et al., 2022	[11C]-PiB	ePET	0-10 min	[18F]-FDG	90	VOI-based correlation: r=~0.70-0.95; voxel-wise analysis
[30]	Chen et al., 2015	[11C]-PiB	R1 (SRTM2)	\	[15O]-H2O	19	VOI-based correlation: ϱ=~0.80-0.90; also for K1: ϱ=~0.50-0.80
[31]	Choi et al., 2023	[18F]-FBB	DL (GAN)	90-110 min	[18F]-FDG	110	SSIM=0.768, PSNR=32.4
[32]	Daerr et al., 2017	[18F]-FBB	ePET	0-5 min / 0-10 min	[18F]-FDG	33	VOI-based correlation: r=0.86; intra-reader agreement: κ=0.79-0.87
[33]	Dghoughi et al., 2019	[18F]-FMM	ePET	0-1 min	[18F]-FDG	19	VOI-based correlation: r=0.76
[34]	Fettahoglu et al., 2024	[18F]-FBB	ePET	0-2 min	[15O]-H2O	20	VOI-based correlation: r=0.90; voxel-wise analysis; optimal timeframe analysis
[35]	Florek et al., 2018	[18F]-FBB	ePET	0-10 min	None	112	Observational; positive correlation with MMSE
[36]	Forsberg et al., 2012	[11C]-PiB	ePET	0-6 min	[18F]-FDG	64	VOI-based correlation: r=~0.39-0.74; correlation with K1: r=0.71
[37]	Fu J. et al., 2025	[18F]-MK-6420	ePET, R1 (SRTM) + K1	0-3 min	[15O]-H2O	17	VOI-based correlation: r=0.84, r=0.88; for K1: ϱ=0.57

[38]	Fu L. et al., 2014	[11C]-PiB	ePET	1.33-8 min	[18F]-FDG	40	VOI-based correlation: r=0.87; optimal timeframe analysis; SS=0.64, SP=0.71
[39]	Gómez-Grande et al., 2023	[18F]-FBP [18F]-FMM	ePET	0-1 min 0-1 min	[18F]-FDG	17	VOI-based correlation: r=0.92
[40]	Guehl et al., 2023	[18F]-MK-6420 [11C]-PiB	R1 (SRTM2)	\	Compared to each other	49	VOI-based correlation: r=0.95
[41]	Hammes et al., 2017	[18F]-FTP	ePET	1-6 min	[18F]-FDG	20	VOI-based correlation: r=~0.82-0.95; voxel-wise correlation: r=0.87; optimal timeframe analysis
[42]	Hsiao et al., 2012	[18F]-FBP	ePET, R1 (SRTM)	0-2 min / 1-6 min	[18F]-FDG	14	VOI-based correlation: r=0.78/0.87, r=0.78; voxel-wise correlation: r=0.92/0.95, r=0.91; optimal timeframe analysis
[43]	Jeong et al., 2019	[18F]-FPN	ePET	0-10 min	[18F]-FDG	33	VOI-based correlation: r=0.83
[44]	Joseph-Mathurin et al., 2018	[11C]-PiB	ePET, R1 (SRTM)	1-9 min	[15O]-H2O (+ [18F]-FDG)	110	Voxel-wise correlation: r=0.71, r=0.74; optimal timeframe analysis
[45]	Kwon et al., 2021	[18F]-FBB	ePET	0-10 min	ECD SPECT	27	VOI-based correlation: r=0.90; voxel-wise analysis; ROC AUC=0.91
[46]	Leuzy et al., 2018	[18F]-THK5317	ePET, R1 (SRTM)	0-3 min	[18F]-FDG	16	VOI-based correlation: r=0.83, r=0.85; voxel-wise analysis
[47]	Lin et al., 2016	[18F]-FBP	ePET	1-6 min	None	82	voxel-wise analysis; correlation with MMSE: r=0.52
[48]	Lojo-Ramírez et al., 2025	[18F]-FBB	ePET	0-5 min	[18F]-FDG	103	VOI-based correlation: ϱ=0.88; voxel-wise analysis; ROC AUC=0.86; intra-reader agreement: κ=0.78
[49]	Matthews et al., 2022	[18F]-FBP	ML	0-6 min	[18F]-FDG	111	Correlation between classifier scores: r=0.90; correlation with MMSE: r=0.61
[50]	Meyer et al., 2011	[11C]-PiB	R1 (SRTM2)	\	[18F]-FDG	22	VOI-based correlation: r=0.79; correlation analysis with MMSE
[51]	Myoraku et al., 2022	[18F]-FBP, [18F]-FBB	ePET	45s-6 min, 45s-6 min	[18F]-FDG	100	VOI-based correlation: r=0.74
[52]	Oliveira et al., 2018	[11C]-PiB	ePET, R1 (MRTM)	0-6 min/ 1-8 min	[18F]-FDG	52	Voxel-wise correlation: r=0.81-0.82, r=0.80-0.83
[53]	Ottoy et al., 2019	[18F]-FBP	ePET, R1 (SRTM2)	0-2 min	[15O]-H2O (+ [18F]-FDG)	39	VOI-based correlation: r=0.70-0.94, r=0.65-0.92; voxel-wise analysis; ROC AUC=0.87-0.95, 0.86-0.95; optimal timeframe analysis; good correlations with FDG and MMSE
[54]	Peretti et al., 2019a	[11C]-PiB	ePET, R1 (SRTM2)	20s-130s/ 1-8 min	[18F]-FDG	30	VOI-based correlation: r=0.76, r=0.85; voxel-wise analysis; optimal timeframe analysis
[55]	Peretti et al., 2019b	[11C]-PiB	ePET, R1 (SRTM2)	20s-130s/ 1-8 min	[18F]-FDG	52	ROC AUC=0.94/0.89, 0.92; correlation of PETscores: r=0.87/0.82, r=0.90
[56]	Peretti et al., 2021	[11C]-PiB	R1 (SRTM2)	\	[18F]-FDG	79	ROC AUC=0.81; disease patterns show a correlation of 0.76
[57]	Peretti et al., 2022	[11C]-PiB	ePET, R1 (SRTM2)	20s-130s/ 1-8 min	[18F]-FDG	52	VOI-based correlation: r=0.59/0.49, r=0.79; ROC AUC=0.69/0.85, 0.83
[58]	Ponto et al., 2019	[11C]-PiB	ePET, R1 (SRTM2)	3.5-4 min/ 0-6 min/ peak	[15O]-H2O	24	VOI-based correlation: r=0.61/0.52/0.64, r=0.62; intrasubject correlation: r=0.78/0.82/0.80, r=0.82

[59]	Ribaldi et al., 2025	[18F]-FBP, [18F]-FMM	ePET	0-5 min, 0-10 min	ASL MRI	46	Significant correlations found through linear regression; also significant differences in SUVR
[60]	Rodriguez-Vieitez et al., 2016	[11C]-PiB	ePET, R1 (SRTM)	1-4 min	[18F]-FDG	41	VOI-based correlation: r=0.61; voxel-wise analysis; ROC AUC=0.84-0.90; optimal timeframe analysis
[61]	Rodriguez-Vieitez et al., 2017	[18F]-THK5317, [11C]-PiB	ePET, R1 (SRTM)	0-3 min, 1-8 min	[18F]-FDG	20	VOI-based correlation: r=0.86/0.88 (eTHK/ePiB), r=0.86/0.90 (r1 THK/ r1 PiB); voxel-wise analysis; ROC AUC=0.82/0.78 (eTHK/ePiB), 0.84/0.79 (r1 THK/ r1 PiB); optimal timeframe analysis
[62]	Rostomian et al., 2011	[11C]-PiB	ePET, R1 (SRTM)	1-8 min	[18F]-FDG	83	VOI-based correlation: r=0.91; voxel-wise correlation: r=0.80; correlation with MMSE: r=0.41
[63]	Sanaat et al., 2024	[18F]-FBP, [18F]-FMM	DL (TNN)	0-5 min, 0-10 min	[18F]-FDG	166	VOI-based correlation: r=0.82, r=0.85; voxel-wise analysis; SSIM=~0.92; PSNR=~30.4
[64]	Schmitt et al., 2021	[18F]-FMM	ePET	0-10 min	[18F]-FDG	20	VOI-based correlation: r=0.86; intra-reader agreement: ICC=0.75-0.86
[65]	Segovia et al., 2018a	[18F]-FBB	ePET	0-10 min	[18F]-FDG	47	VOI-based correlation: r=~0.5; voxel-wise analysis; intra-reader agreement: κ=0.75-0.78
[66]	Segovia et al. 2018b	[18F]-FBB	ML (SVM)	*Not reported*	[18F]-FDG	47	ACC=~0.72, SS=~0.75, SP=~0.70
[67]	Segovia et al., 2020	[18F]-FBB	ML (SVM)	0-20 min	[18F]-FDG	43	ROC AUC>0.8; multiple models/features tested
[68]	Seiffert et al., 2020	[18F]-FBP	ePET	0-10 min	[18F]-FDG	19	VOI-based correlation: r=0.72
[69]	Seiffert et al., 2021	[18F]-FBP, [18F]-FBB, [18F]-FMM	ePET	0-1 min 0-1 min 0-1 min	[18F]-FDG	60	VOI-based correlation: r = 0.86 (FBP), r = 0.77 (FBB), r = 0.78 (FMM); intrapatient correlation: r=0.93
[70]	Son et al., 2020	[18F]-FBB	ePET	0-5 min	[18F]-FDG	40	VOI-based correlation: r=~0.77; intra-reader agreement: κ=~0.82
[71]	Tiepolt et al., 2016	[11C]-PiB, [18F]-FBB	ePET	1-9 min, 1-9 min	[18F]-FDG	22	VOI-based correlation: r=0.73, r=0.81; voxel-wise analysis; correlation with MMSE: ρ=0.46
[72]	Tiepolt et al., 2019	[11C]-PiB	ePET	1-9 min	None	31	Voxel-wise analysis; significant correlations found with cognitive performance
[73]	Tuncel et al., 2023	[18F]-FBP, [18F]-FTP	R1 (SRTM)	\	Compared to each other	50	VOI-based correlation: r=0.89-0.93; voxel-wise analysis
[74]	Vanhoutte et al., 2021	[18F]-FBP	ePET	0-4 min	[18F]-FDG	191	Voxel-wise analysis; optimal timeframe analysis; intrapatient correlation: r=~0.85; associations found with clinical diagnosis/cognition
[75]	Völter et al., 2023	[18F]-PI-2620, [18F]-FMM	ePET	0.5-2.5 min 0-10 min	Compared to each other	64	VOI-based correlation: r=0.82; correlation with cognition: ρ=~0.58
[76]	Völter et al., 2025	[18F]-FBB, [18F]-FMM	ePET	0-10 min 0-10 min	None	82	Voxel-wise analysis; associations found with MMSE
[77]	Wolters et al., 2020	[18F]-FTP	R1 (RPM)	\	[18F]-FDG	133	VOI-based correlation: stβ=0.82-0.92; voxel-wise analysis; ROC AUC=0.77 (DLB <-> AD) /0.94 (DLB <-> HC)

[78]	Yoon et al., 2021	[18F]-FBB	ePET, R1 (SRTM2)	0-10 min	Compared to each other	60	VOI-based correlation: r=0.75-0.91; voxel-wise analysis; positive correlation with MMSE score

Abbreviations: ROC AUC = Receiver Operating Characteristic Area Under the Curve; SS = sensitivity; SP = specificity; ACC = accuracy; SSIM = structural similarity index; SRTM(2) = simplified reference tissue model (2); ML = machine learning; SVM = support vector machine; DL = deep learning; TNN = transformer neural network; GAN = generative adversarial network; VOI = volume of interest; MMSE = mini mental state exam; R1 = relative delivery rate; K1 = influx rate; ePET = early phase PET; AD = Alzheimer's disease; DLB = Lewy-body dementia; ICC = intraclass correlation coefficient; [11C]-PiB = [11C]-Pittsburgh compound B; [18F]-FBB = [18F]-florbetaben; [18F]-FBP = [18F]-florbetapir; [18F]-FMM = [18F]-flutemetamol; [18F]-FPN = [18F]-florapronol; [18F]-FTP = [18F]-flortaucipir; ASL MRI = arterial spin labelling magnetic resonance imaging.
